# Dual Role of Ionic Liquids as Plasticizer and Co-Foaming Agent of Polylactide Matrix

**DOI:** 10.3390/polym17222967

**Published:** 2025-11-07

**Authors:** Debora P. Schmitz, Luanda Lins, Juliana M. Farias da Silva, Bluma G. Soares, Sebastien Livi

**Affiliations:** 1 Instituto de Engenharias Integradas, Universidade Federal de Itajubá, Itabira 37500-903, Minas Geiras, Brazil; debora.p.schmitz@unifei.edu.br; 2 Universite Claude Bernard Lyon 1, INSA Lyon, Université Jean Monnet, CNRS, UMR 5223, Ingénierie des Matériaux Polymères, F-69621 Villeurbanne cédex, France; 3 Universidade Federal do Rio de Janeiro, PEMM-COPPE, Centro de Tecnologia, Rio de Janeiro 21941-594, Brazil

**Keywords:** poly(lactic acid), foams, supercritical CO_2_, ionic liquid, biopolymer

## Abstract

Polylactic acid (PLA) is considered as an attractive polymer due to its renewable origin, biodegradability, and promising tensile strength and modulus. However, its inherent brittleness, characterized by a low impact resistance and elongation at break, can significantly restrict its application. This work proposes a new insight to improve the toughness of PLA while keeping its biocompatibility by incorporating two biocompatible ionic liquids (ILs), 1-ethyl-3-methylimidazolium ethyl sulfate ([emim][EtSO_4_]), and tris(2-hydroxyethyl) methylammonium methylsulfate ([Tris][MeSO_4_]). The modified PLA systems were thoroughly characterized to evaluate their mechanical and thermal behavior. Results demonstrated that the addition of 1 wt% of either IL resulted in significant improvement in modulus. Increasing the amount of IL resulted in an increase in the toughness while maintaining the material’s original stiffness and also the thermal stability. Furthermore, the foaming potential of the modified PLA using supercritical CO_2_ was investigated as an environmentally friendly processing method. The ionic liquids contributed positively to the foamability of the material, suggesting improved gas solubility and cell nucleation during the foaming process. The addition of both IL decreased the cell size and resulted in narrower cell size distribution. These findings highlight the potential of ionic liquid-modified PLA systems for the processing of lightweight, and high-performance packaging materials.

## 1. Introduction

Poly(lactic acid) (PLA) is one of the most widely used bio-based thermoplastics, extensively applied in packaging, particularly food packaging [1], as well as in various consumer products, and biomedical and pharmaceutical applications. Its widespread adoption is largely due to its unique properties, including biodegradability, biocompatibility, high tensile strength, and a relatively high elastic modulus [2,3]. Additionally, PLA is commercially available at a relatively low-cost compared to other biodegradable polymers. Despite these advantages, PLA suffers from several limitations, such as low thermal stability, low melt strength, and inherent brittleness, which hinder its processability and restrict its use in more demanding applications. To address these drawbacks, especially its poor toughness, numerous strategies have been explored these last years. These include blending with more ductile polymers [4,5,6,7], such as poly(butylene adipate-co-terephthalate) (PBAT), ethylene—vinyl acetate (EVA) copolymers, polypropylene (PP), poly(butylene succinate) (PBS), and poly(butylene succinate-co-adipate) (PBSA), as well as incorporating plasticizing agents, especially those derived from renewable resources, such as epoxidized vegetable oils [8,9,10,11].

In recent years, ionic liquids (ILs) have emerged as promising additives for a variety of polymer systems due to their distinctive properties, including negligible volatility, moderate viscosity, good thermal stability, and structural tunability [12,13,14]. ILs have been studied as plasticizers in various polymer matrices, and their non-volatile nature is particularly advantageous, as it reduces the likelihood of migration or leaching from the material [13]. However, despite the potential of ILs, studies investigating their role as plasticizers and toughening agents, especially in PLA systems, remain limited. Some notable studies have begun to explore this area. For instance, Chen et al. [15] incorporated 10% and 20% of 1-methyl-3-pentylimidazolium-based IL with different anions, hexafluorophosphate [PF_6_^−^], tetrafluoroborate [BF_4_^−^], and bis(trifluoromethanesulfonyl) imide [TFSI], into PLA matrix via solution processing. Among the tested ILs, the PF_6_^−^-based IL demonstrated the most significant improvements in both ductility and thermal stability. Similarly, Jia et al. [16] melt-blended 3% tetrabutylphosphonium [BF_4_^−^] into PLA, resulting in a marked enhancement of crystallization ability of PLA. The elongation at break increased from 8.5 to 222%, and the impact resistance doubled. Gui et al. [17] used a functional IL, 3-methyl-1-(ethoxycarbonylethyl) imidazolium [BF_4_^−^], in PLA blends via melt processing. The addition of IL led to reductions in both glass transition temperature (Tg) and cold crystallization temperature (T_cc_), highlighting their plasticizing effect. Mechanical testing revealed substantial increases in elongation at break and impact strength, with optimal performance observed at 7% IL content. Park et al. [18,19] investigated the influence of two alkyl phosphonium-based ILs (with decanoate and tetra fluoroborate anions) on PLA. Melt-blending with 10% IL containing decanoate resulted in a 15 °C reduction in Tg, whereas BF_4_^−^-based IL caused a 5 °C decrease. Zhang et al. [20] used 1-butyl-3-methyl imidazolium [PF_6_^-^] (bmim.PF6) as a plasticizer for PLA via solvent casting. Tg dropped from 58 °C to 55 °C with 2% IL, and by more than 18 °C at 10% IL loading. All blends remained transparent, indicating good miscibility between the IL and PLA. More recently, Merlini et al. [21] synthesized epoxidized imidazolium-based ILs and melt-blended them with PLA. The tri- and tetra epoxidized ILs acted as a chain extender, evidenced by an increase in PLA’s molar mass.

For medical and pharmaceutical applications, both the polymer matrix and the additives must be biocompatible. In this context, 1-ethyl-3-methylimidazolium ethyl sulfate ([emim][EtSO_4_]) and Tris(2-hydroxyethyl)methylammonium methylsulfate ([Tris][MeSO_4_]) have been recognized for their biocompatibility and have received FDA approval for use in biomedical, pharmaceutical, and food-related applications. Therefore, the present work aims to investigate the effects of these two biocompatible ILs on the key properties of PLA, focusing on their role as toughening and plasticizing effects. The incorporation of 10% of [emim][EtSO_4_] resulted in a significant enhancement of elongation at break, while preserving the high Young’s modulus and maintaining a Tg similar to that of neat PLA. These findings suggest that this IL functions effectively as a toughening agent rather than a conventional plasticizer.

To expand the PLA’s potential applications in the biomedical field, the development of foamed PLA materials is of particular interest, especially for tissue engineering and bone tissue restoration, etc. Among several strategies for developing foamed polymeric materials, the supercritical CO_2_ (scCO_2_) approach is especially promising due to its low cost, non-toxicity, non-flammability, and environmentally friendly nature. The literature covers several aspects of PLA foaming using scCO_2_ and highlights the impact of various parameters and additives on foam morphology and properties [22,23,24,25,26]. However, to the best of the authors’ knowledge, no prior studies have explored the use of biocompatible ILs as toughening agents in PLA and their influence on the scCO_2_ foaming process. Therefore, the motivation behind this work was to develop new PLA-based materials by combining biodegradability, biocompatibility of polymer matrices and the ILs with a green, non-toxic foaming process. These materials are envisioned for advanced biomedical applications, particularly in tissue engineering scaffolds.

## 2. Materials and Methods

### 2.1. Materials

Poly(lactic acid), PLA 2002D (1.5–2% of D isomer; density of 1.24 g cm^−3^) was obtained from NatureWorks^®^ LLC Co., Ltd. (Plymouth, MN, USA). Two ILs based on ammonium and imidazolium cations, denoted Tris (2-hydroxyehyl) methylammonium methylsulfate, ([Tris][MeSO_4_]) and 1-ethyl-3 methylimidazolium ethyl sulfate ([emim][EtSO_4_]) were purchased by Sigma Aldrich, as indicated in Table 1.

### 2.2. Blend Preparation

Before extrusion, PLA was dried at 60 °C for 24 h. Polymer/IL blends were prepared using a 15 g-capacity DSM micro-extruder (Midi 2000 Heerlen, NL) with co-rotating screws (length-to-diameter (L/D) ratio: 18). The blends were processed at 150 °C for 3 min under a 70 rpm speed and injected in a 10 cm^3^ mold at 30 °C to obtain dumbbell-shaped specimens.

### 2.3. Foaming Procedure

Based on a previous study [27], we have determined the key parameters, i.e., temperature and pressure governing the foaming of PLA in function of the crystallization under supercritical CO_2_. Thus, we have used the same conditions of foaming of the PLA matrix, and we have studied the influence of the ionic liquid under these same conditions. For these reasons, PLA and PLA/IL samples with dimensions of 30 mm × 12 mm × 3 mm were placed in an autoclave (Model 4565C, Paar Instrument) with a volume of 300 cm^3^. Prior to all experiments, a CO_2_ purge was conducted. In the first step, PLA and PLA/IL blends were saturated with CO_2_ for 1 h at a temperature of 165 °C and a pressure ranging from 10.6 MPa up to 15.2 MPa, depending on the target pressure for the foaming step. This was followed by thermal stabilization, consisting of an isothermal holding period of around 20 min, before initiating polymer expansion. Finally, foaming of the samples was triggered by a rapid pressure drop to atmospheric pressure at a rate of about 20 bar s^−1^. As a result, the neat PLA and PLA/IL blends became supersaturated with CO_2_, leading to cell nucleation and growth.

### 2.4. Characterization

Mechanical properties were evaluated using uniaxial tensile tests, conducted on an MTS 2/M electromechanical testing system at 22 ± 1 °C and 50 ± 5% relative humidity, with a crosshead speed of 5 mm min^−1^. A minimum of five tensile specimens was tested for each reported value. The tensile tests were performed on dumbbell-shaped specimens (AFNOR H3 standard) with a thickness of 2 mm. Prior to testing, the samples were conditioned for 24 h under the same environmental conditions as the tests.

Dynamic mechanical analysis (DMA) was performed using a Q800 dynamic mechanical analyzer (TA Instruments Inc., New Castle, Delaware, USA) equipped with a tension: film clamp. The samples were thermo-pressed into films with thickness of approximately 0.5 mm. The tests were conducted at a frequency of 1 Hz and a strain amplitude of 0.1%, with a heating rate of 3 °C min^−1^ from 25 °C to 120 °C.

Differential scanning calorimetry (DSC) was carried out using a Netzsch 204-F1 instrument (Selb - Germany). The thermal protocol included a first heating scan from 0 to 180 °C at 10 °C min^−1^, followed by a cooling scan at the same rate (10 °C min^−1^) down to 0 °C, and a second heating scan up to 180 °C at 10 °C min^−1^. The degree of crystallinity was estimated using Equation (1) [22]:

(1) $${Χ}_{c}(\%)=\;\frac{{\Delta H}_{m}-{\Delta H}_{cc}}{93.6\;J/g}\;\times 100$$

where ΔH_m_ and ΔH_cc_ are the enthalpies of melting and cold crystallization process, respectively, and 93.6 J/g corresponds to the fusion enthalpy (${∆}_{m}^{0}$) for 100% crystalline PLA [28].

Scanning electron micrograph of the materials was conducted on a TESCAN (Vega 3 model, Brno, Czech Republic) operating at 10 kV. The samples were cryogenically fractured and the surface was sputtered with thin layer of gold before analysis.

Rheological properties were obtained on a Discovery DHR 1 hybrid rheometer from TA Instrument Inc., operating in an oscillatory mode. The analyses were performed using parallel plate geometry of 25 mm diameter, gap of 1.0 mm, at 170 °C in the frequency range from 0.1 to 100 Hz. The amplitude of 0.1% was employed, which is within the linear viscoelastic region.

### 2.5. Foaming Properties

The density of the solid and foamed samples was assessed on a helium picnometer of Micromeritics AccuPyc 133Q V2.03 N at 25 °C. The volume extension ratio (VER) was calculated using Equation (2):

(2) $$VER=\;\frac{\rho_{s}}{\rho_{f}}$$

where *ρ_S_* and *ρ_f_* are the density of the solid and foamed sample, respectively.

The cell density (N_0_) of the foamed samples was determined according to Equation (3):

(3) N0= nA32 ×ρSρf

in which *n* represents the cells number of within statistical area obtained by SEM, and *A* is the statistical area (cm^2^).

## 3. Results

### 3.1. Mechanical and Thermal Properties

The effects of the ILs on the mechanical properties of the PLA matrix are summarized in Table 2. The incorporation of 1% of either IL had no noticeable impact on the elongation at break. However, it led to a significant increase in Young’s modulus. This behavior suggests a strong interaction between PLA and the ILs, resulting in effective reinforcement. In contrast, the addition of 10% ILs caused a reduction in modulus, likely due to the higher concentration of low molar mass compounds acting as plasticizers. Despite this decrease, the modulus values remained comparable to that of neat PLA. Notably, elongation at break increased significantly, indicating enhanced toughness. This effect was more pronounced with the use of [emim][EtSO_4_] IL, suggesting a more effective interaction between PLA and this particular IL. Such interactions are likely facilitated by hydrogen bonding and dipole–dipole forces, which improve interfacial adhesion between polymer chains and reduce crack propagation during tensile testing.

**Figure 1:**
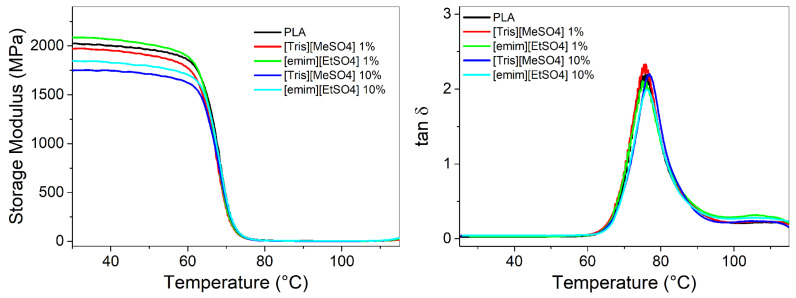
Dynamic mechanical data of PLA blends containing different ionic liquids.

Dynamic-mechanical analysis (DMA) is a powerful technique for evaluating the reinforcing effects of ILs. Figure 1 presents the DMA results in terms of storage modulus and tan delta. The glass transition temperature was determined from the peak maximum of the tan delta curve. The key DMA parameters are also summarized in Table 3. The system containing 1% of [emim][EtSO_4_] showed a slightly higher E’ value in the glassy region compared to neat PLA, which aligns with the observed tensile properties. Increasing the IL content to 10% led to a reduction in modulus, attributed to the lubricating/plasticizing effect of the ILs. However, at both IL concentration (1% and 10%), PLA blends containing [emim][EtSO_4_] exhibited higher modulus values than those with the other IL, suggesting a stronger interaction between PLA and this specific IL, probably through the acidic hydrogen in the C2-position of the imidazolium ring and the ester groups along the PLA chain.

Despite the low molar mass of the ILs, the Tg of the PLA matrix remained unchanged, even at a concentration of 10% showing that ILs do not act as plasticizers but mainly as toughening agents. Typically, compounds that improve PLA toughness, such as epoxidized vegetable oils [29], also reduce stiffness and Tg. Therefore, the ability to enhance toughness without compromising stiffness or thermal transitions is particularly valuable from the technological perspective.

**Figure 2:**
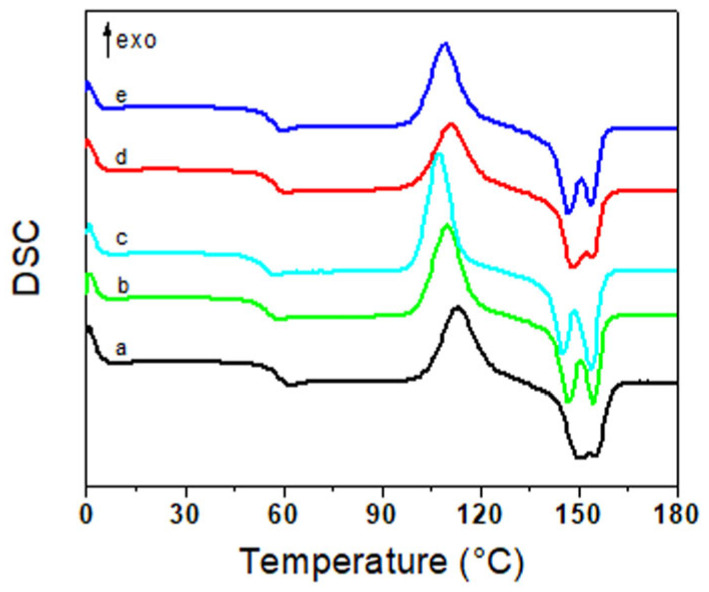
Second heating scan obtained from DSC analysis for (a) neat PLA and the PLA blends containing (b) 1% [emim][EtSO_4_]; (c) 10% [emim][EtSO_4_]; (d) 1% [Tris][MeSO_4_]; (e) 10% [Tris][MeSO_4_].

DSC was employed to investigate the crystallization and melting behavior of PLA as a function of IL type and content. Figure 2 presents the second heating scans of the samples, highlighting the influence of both the nature and concentration of the ILs. The key thermal parameters associated with the crystallization and melting processes are summarized in Table 3. No distinct crystallization peaks were observed during the non-isothermal cooling process likely due to the inherently slow crystallization rate of PLA. During the second heating cycle, all samples exhibited a glass transition (Tg) in the range of 56–59 °C. The incorporation of ILs resulted in a slight decrease in Tg values. However, this reduction does not imply miscibility between PLA and the ILs. This interpretation is supported by DMA results and SEM microscopy, which also indicate immiscibility between the components. Neat PLA exhibited a cold crystallization exotherm (T_cc_) at approximately 115 °C. Upon IL addition, T_cc_ shifted to lower temperatures, suggesting that the ILs may accelerate the crystallization process of PLA. This effect was more pronounced in the sample containing 10% [emim][EtSO_4_]. All systems displayed two distinct melting endotherms. The first melting peak (T_m1_), appearing in the range of 145–150 °C, is attributed to the melting of crystals formed during the melt-recrystallization process and was only slightly affected by IL addition. The second melting peak (T_m2_) corresponds to the melting of more stable PLA crystals and is consistent with literature reports related to the melting of the PLA chains [30,31]. Although ILs were found to accelerate the crystallization process, they did not enhance the overall crystallinity of PLA. In fact, all IL-containing systems showed a reduction in crystallinity, likely due to interactions between the ILs and PLA chains, which hinder effective chain packing and crystal formation.

### 3.2. Morphology

**Figure 3:**
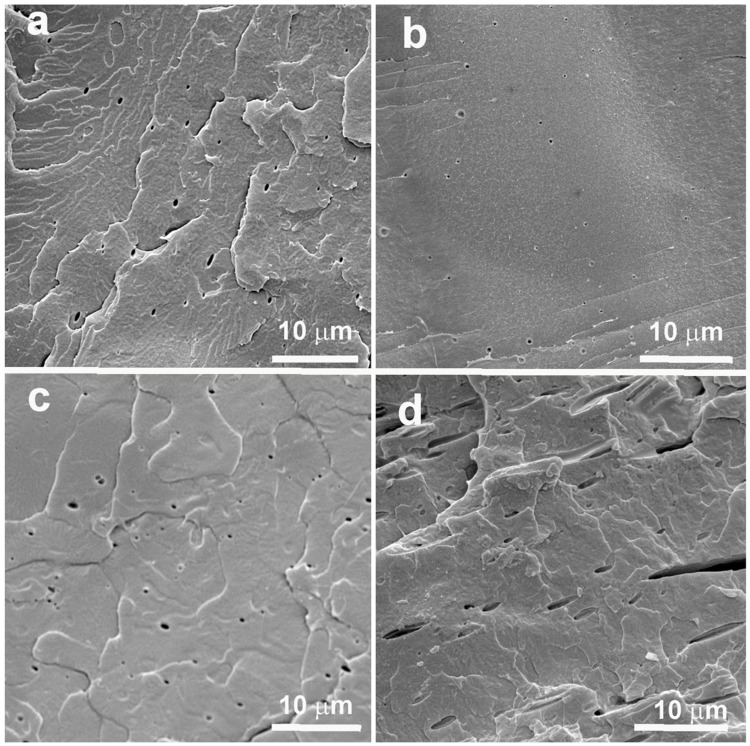
SEM images of PLA containing (**a**) 1% [emim][EtSO_4_]; (**b**) 10% [emim][EtSO_4_]; (**c**) 1% [Tris][MeSO_4_]; (**d**) 10% [Tris][MeSO_4_].

The dispersion of various ILs within the PLA matrix was examined using SEM microscopy, as shown in Figure 3. The morphology of PLA containing 1% of each IL is characterized by the presence of small dispersed IL-domains, confirming the immiscibility of these ILs with the PLA matrix. These morphological observations are consistent with the thermal (Tg) and mechanical (modulus) behavior discussed previously. Among the systems analyzed, the PLA/[emim][EtSO_4_] sample exhibited slightly smaller domain size, suggesting improved dispersion within the PLA matrix compared to the other ILs. In contrast, the PLA/[Tris][MeSO_4_] (10%) system displayed larger, elongated IL domains, indicating poor compatibility and limited dispersion. The PLA/[emim][EtSO_4_] (10%) blend showed a distinct sea-island morphology, with well-defined phase-separated spherical domains of approximately 1 µm in diameter, characteristic of immiscible but compatible system. Interestingly, these spherical voids resemble voids observed in toughened polymer systems containing liquid rubber, which undergo internal cavitation under stress [32]. This microstructural feature likely contributes to the enhanced elongation at break observed in this system. The presence of such cavitated domains suggests good interfacial adhesion between [emim][EtSO_4_] and the PLA matrix, enabling effective energy dissipation during deformation.

### 3.3. Rheological Properties

**Figure 4:**
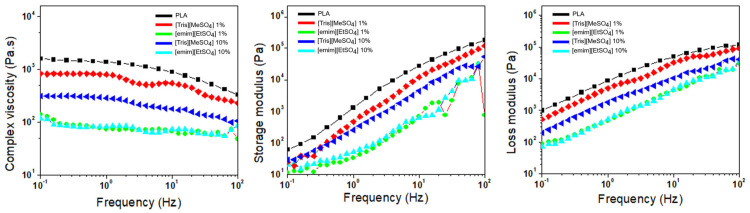
Rheological properties as a function of the frequency for neat PLA and the PLA blends containing different IL content.

The foaming behavior of polymeric materials is strongly governed by their melt elasticity, which is typically assessed by the storage modulus (G′). Figure 4 presents complex viscosity (η*), storage (G′), and loss (G″) moduli against frequency of PLA blends containing various ILs. The incorporation of ILs led to a decrease in both complex viscosity and viscoelastic moduli (G′ and G″), which can be attributed to the plasticizing/lubricating effect imparted by these ILs. Among the studied systems, the PLA/[emim][EtSO_4_] blends exhibited the lowest viscosity values, regardless of IL concentration. This consistent reduction suggests a more favorable interaction between [emim][EtSO_4_] and the PLA matrix, facilitating greater chain mobility and melt flow. The improved compatibility inferred from rheological behavior is consistent with the previously observed morphological and thermal results.

### 3.4. Effect of Ionic Liquids on the Characteristics of Foamed PLA

**Figure 5:**
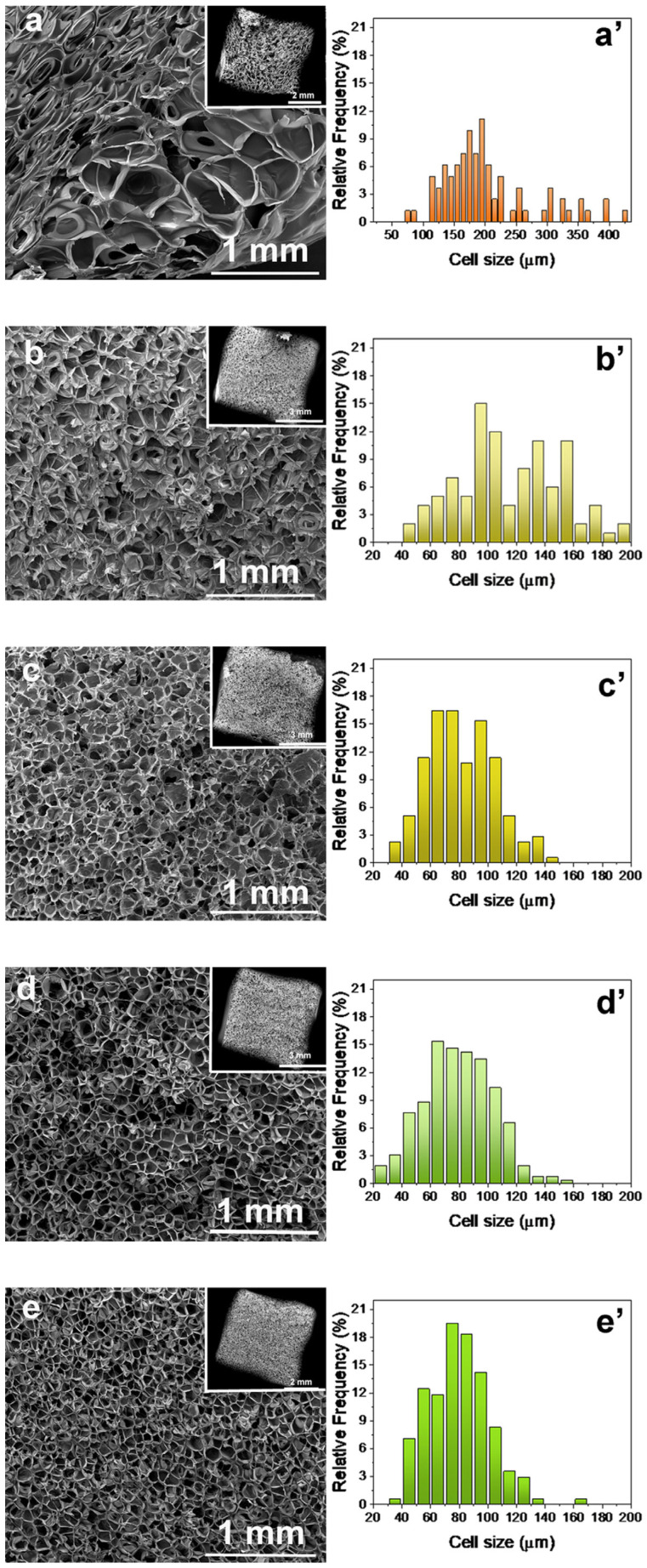
SEM images and cell size distribution obtained from SEM images for the (**a**,**a’**) neat PLA, and their blends containing (**b**,**b’**) 1% [emim][EtSO_4_]; (**c**,**c’**) 10% [emim][EtSO_4_]; (**d**,**d’**) 1% [Tris][MeSO_4_]; and (**e**,**e’**) 10% [Tris][MeSO_4_].

The cell morphology and cell size distribution of the PLA-based foams, obtained from SEM images of cryo-fractured surfaces, are presented in Figure 5. Neat PLA exhibits a heterogeneous cellular structure, with significant variation in cell size from the outer surface toward the interior of the sample. Notably, larger cells are observed near the outer region (right side of the image), while smaller cells are found closer to the core (left side of the image). Moreover, extensive cell collapse and coalescence are evident, resulting in a broad cell size distribution, as shown in Figure 5a This behavior is attributed to the inherently low melt strength of PLA, which limits its ability to withstand the stresses associated with cell nucleation and growth during the foaming process [26]. Consequently, the matrix fails to stabilize the expanding cells, leading to premature cell rupture, coalescence, and poorly developed cellular architecture.

In contrast, the incorporation of ILs leads to a more homogeneous cellular structure, indicating improved foamability, as illustrated in Figure 5b–e. The foams exhibited more uniform cell sizes and narrower cell size distributions, which are further confirmed by the Gaussian-like cell size distribution profiles shown in Figure 5b–e. The most uniform cellular morphologies were observed in PLA blends containing 1% [Tris][MeSO_4_] and 10% of either ILs. According to the literature, Ramdin et al. have demonstrated that [BMIM][MeSO4] have a better CO_2_ solubility of phosphonium combined with the same counter anion [TBMP][MeSO_4_] but less than the corresponding ammonium salt denoted [N1114][MeSO_4_] [33]. These results were confirmed by Jacquemin et al. highlighting the influence of the cations [34]. Thus, ammonium ILs has a better solubility effect compared to imidazolium ILs. However, it is well known that the presence of some functional groups such as hydroxyl, nitrile, methyl, alkyl, or ether group presented on the cation lead to a significant decrease in the CO_2_ solubility [35]. These results can explain the reduction in the cell size by adding [emim][EtSO_4_] in PLA matrix combined with the decrease in viscosity helping for the PLA foaming.

Table 4 summarizes the main cellular parameters of the foamed PLA systems. The addition of 1% and 10% of [Tris][MeSO_4_] resulted foams with smaller average cell size and significantly higher cell densities compared to neat PLA. A similar trend was observed for the PLA blend containing 10% of [emim][EtSO_4_]. Since these blends presented lower viscosity and lower crystallinity than neat PLA, this behavior is likely due to the interaction between CO_2_, the ILs, and the PLA matrix thus facilitating enhanced gas solubility, resulting in the formation of a more uniform and stable cellular structure.

The DSC profiles of the solid and foamed PLA blends are compared in Figure S1 of the Supporting Information, and the key thermal properties are summarized in Table 5. For neat PLA and blends containing 1 wt% IL, an increase in the degree of crystallinity was observed after the scCO_2_ foaming process, indicating that CO_2_ acted as a crystallization promoter for the PLA matrix. In contrast, blends containing higher IL concentrations exhibited a decrease in crystallinity following foaming. This suggests that, at elevated IL contents, the plasticizing effect of the ILs may interfere with CO_2_-induced crystallization, likely by disrupting chain packing and hindering crystal formation.

## 4. Conclusions

In conclusion, this work highlights the key role of ILs to be used as structuring and toughening agents in a biobased matrix such as PLA. In fact, the incorporation of two FDA-approved ILs denoted 1-ethyl-3-methylimidazolium ethyl sulfate ([emim][EtSO_4_]) and tris(2-hydroxyethyl)methylammonium methylsulfate ([Tris][MeSO_4_]) led to significant increases in strain at break (8.8 to 42 times higher) while maintaining a stiffness similar to or even superior to the neat PLA matrix. Compared to the literature, the use of 10% of ILs does not induce a reduction in the glass transition temperature and premature degradation of the PLA matrix opening a new route for the development of high-performance biobased materials for packaging applications. Moreover, ILs were also used as co-foaming agents of PLA blends leading via supercritical CO_2_ to homogenous PLA/IL foams with a diameter size of about 80 μm compared to 200 μm for the neat PLA. Thus, the combination of CO_2_ and the presence of ILs contained in PLA matrix induces enhanced gas solubility, resulting in the formation of a more uniform and stable cellular structure. This latest result opens up promising perspectives in the development of foam with dimensional stability for potential tissue regeneration applications where PLA is a perfect candidate serving as scaffolds due to its biocompatibility.

## Figures and Tables

**Table 1:** Structure and supplier of ionic liquids.

Ionic Liquids	Chemical Structure	Supplier
Tris (2-hydroxyethyl) methylammonium methylsulfate ([Tris][MeSO_4_])	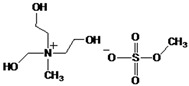	Sigma Aldrich
1-ethyl-3-methylimidazolium ethyl sulfate ([emim][EtSO_4_])	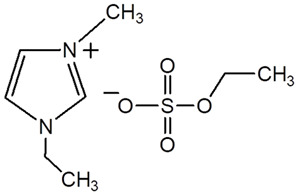	Sigma Aldrich

**Table 2:** Effect of ILs on the mechanical properties of PLA matrix.

IL		Young’s Modulus (MPa)	Strain at Break (%)	Max Stress (MPa)
Type	Weight (%)			
-	-	3158 ± 293	4.2 ± 0.4	80.9 ± 0.6
[Tris][MeSO_4_]	1	3731 ± 257	3.1 ± 0.5	85.9 ± 2.9
[Tris][MeSO_4_]	10	3228 ± 248	36.9 ± 15.0	52.3 ± 13.5
[emim][EtSO_4_]	1	3700 ± 124	3.2 ± 0.4	82.7 ± 1.3
[emim][EtSO_4_]	10	3227 ± 87	177.0 ± 4.5	43.4 ± 2.5

**Table 3:** Effect of ILs on thermal and dynamic-mechanical properties of PLA matrix.

Ionic Liquid		DMA Data		DSC Data						
Type	Weight (%)	E’at 40 °C (MPa)	Tg (°C)	Tg (°C)	T_cc_ (°C)	T_m1_ (°C)	T_m2_ (°C)	ΔH_m_ (J/g)	ΔH_cc_ (J/g)	X_c_ (%)
-	-	2000	75	59	113	150	154	25	20	5.3
[Tris][MeSO_4_]	1	1950	75	58	111	148	154	21	20	1.1
[Tris][MeSO_4_]	10	1750	77	57	109	147	154	24	21	3.2
[emim][EtSO_4_]	1	2070	75	56	110	147	154	24	22	2.1
[emim][EtSO_4_]	10	1850	76	56	107	145	154	24	23	1.1

**Table 4:** Main characteristics of the foamed PLA blends.

Ionic Liquid		Density (g/cm^3^)		VER	Average Cell Size (μm)	Cell Density (10^9^ Cells/cm^3^)
Type	Weight (%)	Solid	Foamed			
-	-	1.2524 ± 0.0005	1.1236 ± 0.0120	1.12	202 ± 74	0.103
[Tris][MeSO_4_]	1	1.2459 ± 0.0005	1.2027 ± 0.0010	1.04	79 ± 24	3.666
[Tris][MeSO_4_]	10	1.1936 ± 0.0010	0.9804 ± 0.0150	1.21	80 ± 21	3.761
[emim][EtSO_4_]	1	1.2546 ± 0.0008	1.1505 ± 0.0100	1.08	114 ± 36	0.562
[emim][EtSO_4_]	10	1.2299 ± 0.0002	1.0361 ± 0.0050	1.18	81 ± 23	2.822

**Table 5:** Main parameters obtained from DSC analysis for the solid and foamed PLA blends.

Ionic Liquid		ΔH_m_ (J/g)		ΔH_cc_ (J/g)		X_c_ (%)	
Type	Weight (%)	A	B	A	B	A	B
-	-	25	23	20	17	5.3	6.4
[Tris][MeSO_4_]	1	21	27	20	21	1.1	6.4
[Tris][MeSO_4_]	10	24	24	21	23	3.2	1.1
[emim][EtSO_4_]	1	24	30	22	25	2.1	5.3
[emim][EtSO_4_]	10	24	30	23	29	1.1	1.1

A: Solid sample; B: foamed sample; ΔH_m_ = heat of fusion; ΔH_cc_ = enthalpy of cold crystallization; X_c_ = degree of crystallinity.

## Data Availability

The datasets generated during and/or analyzed during the current study are available from the corresponding author on reasonable request.

## Supplementary Materials

The following supporting information can be downloaded at: https://www.mdpi.com/article/10.3390/polym17222967/s1, Figure S1: DSC profile related to the second heating scan for solid and foamed PLA blends.
