# Supplementary material for: Dual Role of Ionic Liquids as Plasticizer and Co-Foaming Agent of Polylactide Matrix
Source: Polymers (Basel). 2025 Nov 7;17(22):2967. doi: 10.3390/polym17222967 (PMC12656597; doi:10.3390/polym17222967)
Supplement: Supplementary file 1 [file polymers-17-02967-s001.zip › polymers-3928666-supplementary.pdf]

Supporting Information

Figure S1. DSC profile related to the second heating scan for solid and foamed PLA blends

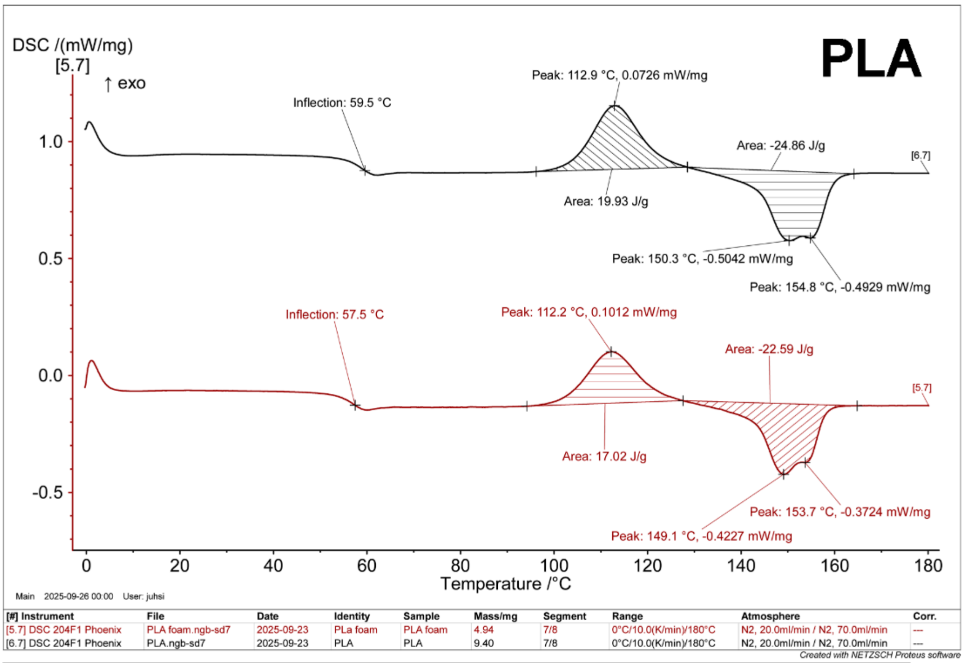

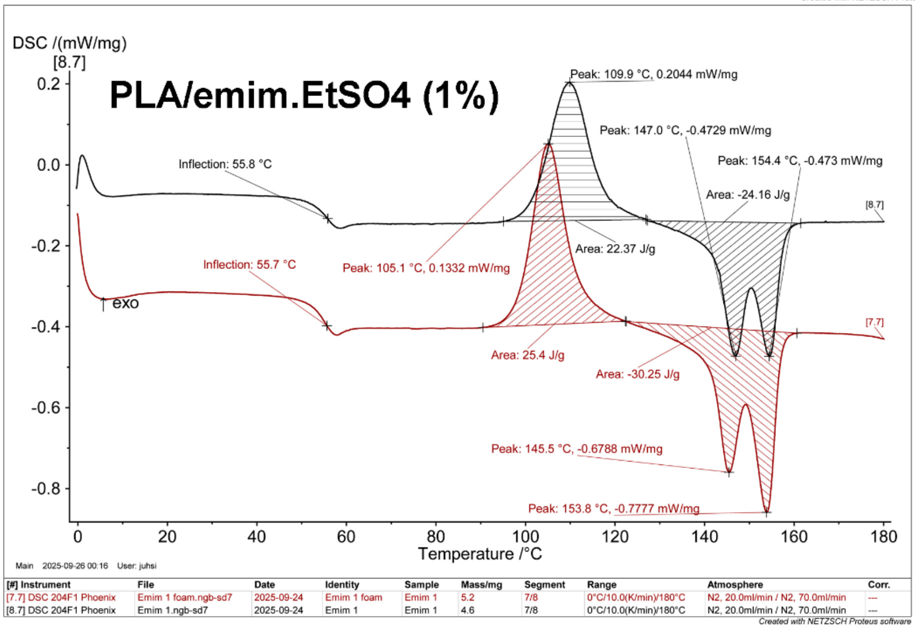

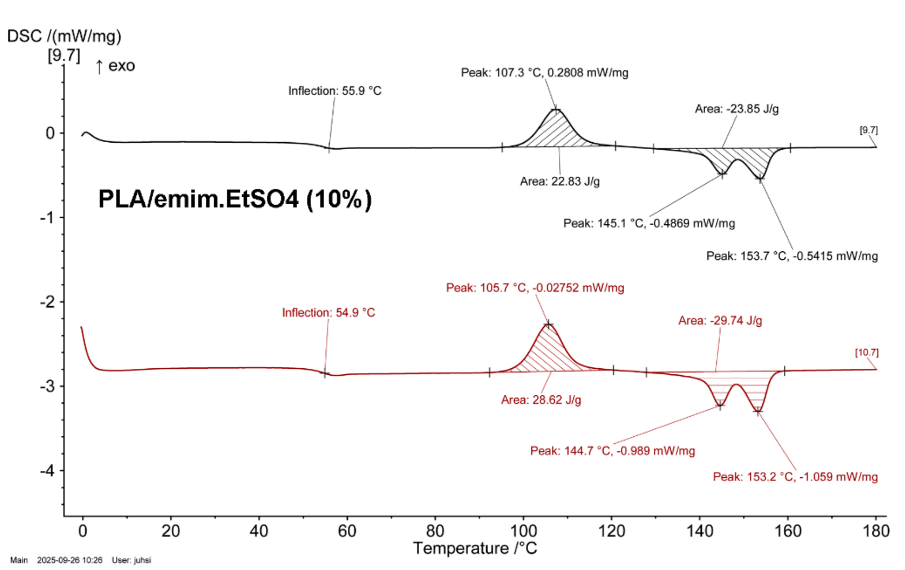

[#] Instrument	File	Date	Identity	Sample	Mass/mg	Segment	Range	Atmosphere	Corr.
[9.7] DSC 204F1 Phoenix	Emim 10 .ngb-sd7	2025-09-24	Emim 10	Emim 10	8.64	7/8	0°C/10.0(K/min)/180°C	N2, 20.0ml/min / N2, 70.0ml/min	---
[10.7] DSC 204F1 Phoenix	Emim 10 foam.ngb-sd7	2025-09-23	Emim 10 foam	Emim 10 foam	4.4	7/8	0°C/10.0(K/min)/180°C	N2, 20.0ml/min / N2, 70.0ml/min	---

Created with NETZSCH Proteus software

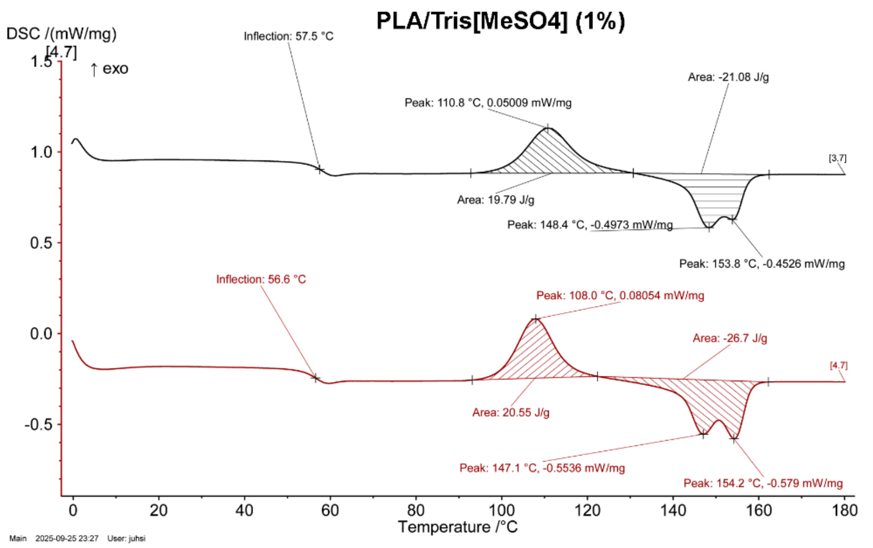

[#] Instrument	File	Date	Identity	Sample	Mass/mg	Segment	Range	Atmosphere	Corr.
[3.7] DSC 204F1 Phoenix	Tris 1.ngb-sd7	2025-09-25	Tris 1	Tris 1	7.8	7/8	0°C/10.0(K/min)/180°C	N2, 20.0ml/min / N2, 70.0ml/min	---
[4.7] DSC 204F1 Phoenix	Tris 1 foam.ngb-sd7	2025-09-25	Tris 1 foam	Tris 1 foam	6.2	7/8	0°C/10.0(K/min)/180°C	N2, 20.0ml/min / N2, 70.0ml/min	---

Created with NETZSCH Proteus software

PLA/Tris[MeSO4] (10%)

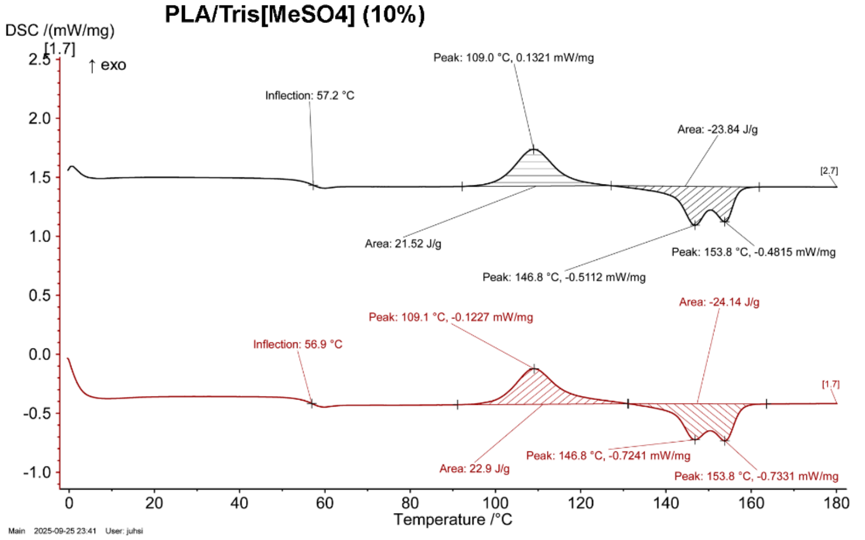

[#] Instrument	File	Date	Identity	Sample	Mass/mg	Segment	Range	Atmosphere	Corr.
[1.7] DSC 204F1 Phoenix	Tris 10 foam.ngb-sd7	2025-09-24	Tris 10 foam	Tris 10 foam	6.4	7/8	0°C/10.0(K/min)/180°C	N2, 20.0ml/min / N2, 70.0ml/min	---
[2.7] DSC 204F1 Phoenix	Tris 10.ngb-sd7	2025-09-24	Tris 10	Tris 10	5.7	7/8	0°C/10.0(K/min)/180°C	N2, 20.0ml/min / N2, 70.0ml/min	---

Created with NETZSCH Proteus software
